# Intersexual Trophic Niche Partitioning in an Ant-Eating Spider (Araneae: Zodariidae)

**DOI:** 10.1371/journal.pone.0014603

**Published:** 2011-01-27

**Authors:** Stano Pekár, Martina Martišová, Trine Bilde

**Affiliations:** 1 Department of Botany and Zoology, Faculty of Sciences, Masaryk University, Brno, Czech Republic; 2 Department of Biological Sciences, Aarhus University, Aarhus, Denmark; University of Plymouth, United Kingdom

## Abstract

**Background:**

Divergence in trophic niche between the sexes may function to reduce competition between the sexes (“intersexual niche partitioning hypothesis”), or may be result from differential selection among the sexes on maximizing reproductive output (“sexual selection hypothesis”). The latter may lead to higher energy demands in females driven by fecundity selection, while males invest in mate searching. We tested predictions of the two hypotheses underlying intersexual trophic niche partitioning in a natural population of spiders. *Zodarion jozefienae* spiders specialize on *Messor barbarus* ants that are polymorphic in body size and hence comprise potential trophic niches for the spider, making this system well-suited to study intersexual trophic niche partitioning.

**Methodology/Principal Findings:**

Comparative analysis of trophic morphology (the chelicerae) and body size of males, females and juveniles demonstrated highly female biased SSD (Sexual Size Dimorphism) in body size, body weight, and in the size of chelicerae, the latter arising from sex-specific growth patterns in trophic morphology. In the field, female spiders actively selected ant sub-castes that were larger than the average prey size, and larger than ants captured by juveniles and males. Female fecundity was highly positively correlated with female body mass, which reflects foraging success during the adult stage. Females in laboratory experiments preferred the large ant sub-castes and displayed higher capture efficiency. In contrast, males occupied a different trophic niche and showed reduced foraging effort and reduced prey capture and feeding efficiency compared with females and juveniles.

**Conclusions/Significance:**

Our data indicate that female-biased dimorphism in trophic morphology and body size correlate with sex-specific reproductive strategies. We propose that intersexual trophic niche partitioning is shaped primarily by fecundity selection in females, and results from sex-differences in the route to successful reproduction where females are selected to maximize energy intake and fecundity, while males switch from foraging to invest in mating effort.

## Introduction

Divergence among the sexes in morphological traits, or in ecology, life history and behavior is widespread in the animal kingdom. Sexual dimorphism in habitat choice or food requirements [1], [2] may lead to intraspecific divergence in the trophic niche, where the sexes differ profoundly either in the diversity and size range of food items, or in the rate of prey capture. The proximate explanations for such trophic divergence between the sexes are diverse and may include differential handling skills [3], developmental size [4], nutritive requirements [5], habitat requirements [6], and availability of suitable prey [7].

The ultimate causes underlying intersexual divergence are sought in two classes of explanations that are not mutually exclusive [6], [8]. The most widely accepted explanation holds that sexual dimorphism results from differential selection among the sexes on traits that confer advantages in reproduction, often referred to as the “sexual selection hypothesis” [8], [9], [10]. Evidence for the sexual selection hypothesis comes primarily from studies on sexual size dimorphism (SSD) where large body size in males confers advantages in male-male competition for females [11], or from studies on color dimorphism for example plumage dimorphism in birds where more ornamented males are more attractive to females [12]. Sexual dimorphism in body size may subsequently result in differential use of resources [13], which may in turn reinforce SSD. The opposite pattern of female-biased SSD arise through fecundity selection on females where large body size results in more offspring [14], subsequently leading to intersexual trophic niche partitioning [15]. The sexual selection and female fecundity hypotheses make similar predictions of a positive relationship between the magnitude of the dimorphic trait and the reproductive benefit associated with the trait [8].

An alternative explanation for the evolution of sex-specific traits is the “intersexual niche partitioning” hypothesis. This hypothesis suggests that sexual dimorphism result from resource competition between individuals, and functions to reduce intersexual competition for habitat or food requirements [16]. The intersexual niche partitioning hypothesis predicts certain sex-specific distribution patterns, but empirical examination of underlying explanations is not straightforward [6], [8]. This is because causal ecological factors are difficult to identify or manipulate. It was proposed that sexual dimorphism in trophic morphology in a direction not predicted by sexual selection would support the intersexual niche partitioning hypothesis [6], [17]. For example, intersexual differences in head size and hence in prey handling and prey selection in lizards was suggested to support the intersexual niche partitioning hypothesis [18]. However, whether head size dimorphism evolved to reduce intersexual competition, or as a male-male competition adaptation remains unresolved, emphasizing that ecological factors may act in concert with other selective forces, or act to reinforce the divergence of sexual dimorphism [6].

Here we tested predictions of the “sexual selection” and “intersexual niche partitioning” hypotheses underlying intersexual trophic niche partitioning in a natural population of spiders. Our study species was *Zodarion jozefienae* (Bosmans), a spider that specializes on polymorphic *Messor barbarus* (Linnaeus) ants. This genus consists of ant-eating spiders that typically associate closely with a single ant species, and different developmental stages of the spiders appear to feed on the same ant species [19]. The prey of *Z. jozefienae* spiders are granivorous *Messor* ants that show considerable polymorphism in body size with a continuous variation which provide potential discrete trophic niches (small, medium and large sized sub-castes) for the different developmental stages and both sexes of the spiders. This predator-prey system is therefore ideally suited for the study of intersexual trophic niche partitioning in the field.

The “intersexual niche partitioning” hypothesis assumes that trophic niche partitioning functions to reduce competition between the sexes. We determined the natural availability and frequency distribution of the different ant size sub-castes, and compared these wiht the preference of males, females and juvenile spiders for ants of different sizes relative to background availability. To examine the evidence for intersexual resource competition, we compared SSD in body size and trophic morphology in juvenile and adult spiders to age-specific and sex-specific trophic niches in the field. The chelicerae are used in prey capture and prey handling, hence we performed comparative analysis of chelicerae morphology, body size and growth trajectories in females, males and juveniles *Z. jozefienae* spiders.

If trophic niche partitioning is driven by fecundity selection [20], we expect female biased SSD in trophic morphology and body size, and a positive relationship between female body size/mass and egg production. Differential routes to maximizing reproduction among the sexes, where females maximize fecundity and males maximize the number of mates [10], are expected to result in higher energetic requirements in females than in males. This would predict differential prey use among the sexes, and sex-specific adaptations in trophic morphology and prey capture efficiency as factors underlying intersexual trophic niche partitioning [21], [22].

## Methods

### SSD in trophic morphology and body size

We characterized morphological traits of adult males, adult females, subadult males, subadult females and juveniles (i.e. immature spiders that could not be identified to sex at the time of capture) of *Zodarion jozefienae*. Subadult males and females were recognized by the presence/absence of swollen palps (males) and the contours of the epigynal plate above the epigastric furrow (females). The material came from pitfall-trap sampling at the study site (given below) prior to the field experiments. Specifically, we measured the width of the prosoma (head), total body length and the length of the basal cheliceral segment (trophic morphology), as the chelicerae are used for prey capture and are expected to be directly related to prey capture efficiency. These morphological structures did not differ in shape between males and females but rather in size [23]. The measurements were taken using an ocular micrometer (accuracy 0.01 mm) of an Olympus SZX9 stereomicroscope.

### Trophic niches in the field

Field experiments were performed in Southern Portugal (Baixo Alentejo) at a pasture (37°37.965′ N, 7°34.191′W) close to Mértola, where only a single species of the genus *Zodarion*, namely *Z. jozefienae*, occurs. This semi-desert area hosts plenty of nests of the prey ant *M. barbarus*. These ants are polymorphic with three worker size classes: minor (<5 mm), media (5–10 mm) and major (>10 mm) [24]. The spiders are associated with the ant nests and forage on the ant trails leading to and from the nest. From June to September the ants as well as the spiders were found to be nocturnal (S. Pekár & M. Martišová, pers. obs.) so observations were made at 20 ant nests between 22.00 and 02.00, when the spiders were most abundant.

To establish the distribution of worker ant size classes we sampled 10 individuals from an ant trail from each of 10 different ant nests. A sample of ants was collected from a random point of the ant trail leading to the nest. The size of the ants (length of ant body) was measured with a ruler to determine ant sub-caste. We examined the spiders' natural prey by following an individual spider for at least 15 min. During the total of 200 census hours we followed 473 individual spiders and recorded 108 prey capture events. The spiders and their ant prey from these events were collected and the body length of the ant prey as well as the body length and the sex of each spider were recorded.

In the field we observed mating in *Z. jozefienae* and collected females with eggsacs from their igloo-shaped silk retreats. Mating in these spiders is very brief (a few seconds) and takes place during hunting near to the ant trials. The eggsacs are attached to stones close to ant nests (Pekár, pers. observ.). The eggsacs were opened and the number of eggs was recorded. Then the size of 10 eggs from each eggsac, the width of female prosoma and female body length were measured using a stereomicroscope. All spiders and ants collected in the field were preserved in 70% ethanol and deposited in the collection of arachnids at the Department of Botany and Zoology, Masaryk University, Brno.

### Prey size preference and capture efficiency

A total of 120 spiders consisting of forty males, forty females and forty juveniles were collected to be used in laboratory experiments of feeding preferences. Spiders were kept in vials (10 mm diameter, 60 mm long) and fed with *M. barbarus* ants, but for 5 days prior to the experiment they were kept without prey. One day prior to experimental trials each spider was placed in a Petri dish (diameter 35 mm) with a filter paper attached to the bottom and fluon layer on the sides to prevent escape of the ant prey. To test the effect of prey size on prey preference and prey capture efficiency one minor (3–5 mm) and one media-major ant sub-caste (8–11 mm) were released sequentially into the Petri dish in random order. Spiders assessed the prey by tapping it with their front legs, and the latency (time duration) until the first tap, the total number of taps, and the latency to the first attack (time between the first mutual encounter and the attack) were recorded. Once both ants had been attacked and immobilized they were placed on opposite sides of the dish to determine the feeding preference of the spider.

In a second experiment, 60 spiders (20 each of males, females and juveniles of *Z. jozefienae*) were collected and used in a similar experimental set-up as in the previous trial. Each individual spider was offered one ant of a variable size. For each trial, the latency to the first attack, the number of attacks and paralysis latency (time between the first attack and complete immobilization, i.e. when an ant could not raise itself after being touched with forceps) were recorded. Each ant was weighed twice, before being released into the Petri dish and again when it had been fed on and discarded by the spider. By subtracting the final weight from the initial weight the mass extracted by the spider during the feeding event was estimated. Female spiders were then weighed and reared for approximately 2 weeks until they produced eggsacs and the relationship between female body mass and clutch size was established.

### Statistical analyses

Data were analyzed using appropriate statistical methods in R [25]. Continuous measurements (ant size, spider sizes, spider mass, ant mass extracted) were a priori assumed to conform to the Gaussian distribution and were therefore analyzed with linear models (linear regression, ANOVA, ANCOVA). Relationship between the size of chelicerae and the size of prosoma was analyzed on a logarithmic scale using linear regression including an offset. For isometric growth the slope of the relationship would be equal to 1, while for allometric growth the change would be significantly different from 1. Data showing heteroscedastic variance were analyzed with Generalized Linear Models (GLM) with Gamma errors and logarithmic link function (GLM-g). Proportions were analyzed using GLM withbinomial error structure and logit link function (GLM-b), or with the Exact binomial test in order to compare the observed proportion with the expected. Linear Mixed-Effects Models (LME) were used to analyze paired comparisons (latency to tap, number of taps, latency to attack between small and large ants within trials). This procedure allows implementation of a variance function in cases when data are heteroscedastic and perform better than standard models when the data are unbalanced [26]. LME were also used in the analysis of nested measurements of eggs within egg sacs. The parameters were estimated from the minimal model obtained by lumping similar factor levels and differences between levels were compared post hoc using treatment contrasts [27]. Confidence intervals were estimated using normal approximation. Means and standard errors are given for all parameters in the text.

## Results

### SSD in trophic morphology and body size

**Figure 1:**
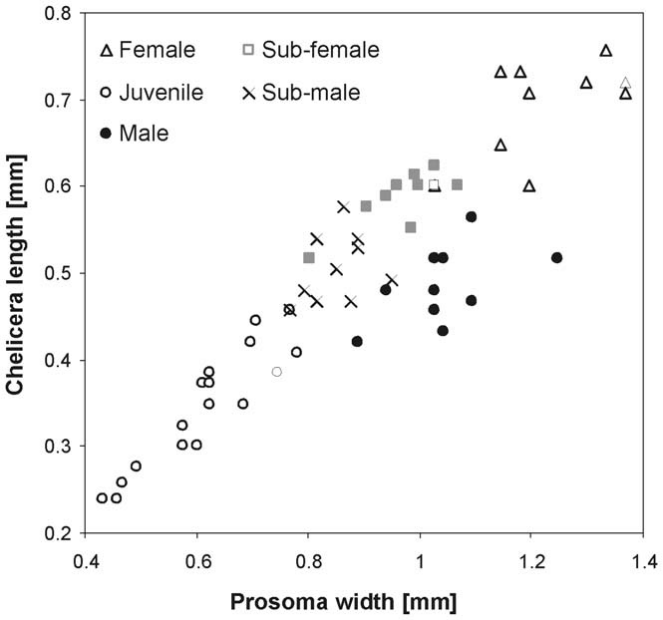
Relationship between the chelicerae length and prosoma width. All stages and both sexes of *Z. jozefienae* are shown with different symbols.

The total body length, prosoma width and the length of chelicerae in adult males of *Z. jozefienae* (Table 1) were significantly smaller/shorter than those of adult females (ANOVA, F_1,18_>49, P<0.0001). The prosoma width of adult males was not significantly different from that of subadult females (contrast, P = 0.5), but chelicerae length of adult males was significantly shorter than that of subadult females (contrasts, P = 0.002). In females, the size of chelicerae correlated with the size of the prosoma (both on logarithmic scale) following a significantly different slope compared with males (ANCOVA, F_3,56_ = 42.2, P<0.0001). While for females the change in the size of chelicerae was isometric (offset regression, P = 0.88), in males the change was negatively allometric (offset regression, P = 0.0001). The chelicerae of subadult males was correlated with prosoma size similarly as in juveniles, while the chelicerae of adult males actually diminished (Table 1, Fig. 1). The ratio of the length of chelicera to the lenght of the prosoma ranged between 56 and 61% for all stages and sexes except for adult males where it was only 47% (Table 1). The weight of males was significantly lower than the weight of females (GLM-g, F_1,16_ = 213, P<0.0001). Gravid females weighed five times more than males (12.6±0.43 *vs*. 2.52±0.69 mg). The sexual dimorphism in favor of females was 18% in prosoma size, 44% in chelicerae size, 49% in total body length and 500% in body mass.

**Table 1:** Overview of the mean (SE) size [mm] of three morphological characteristics in juvenile, subadult and adult stages of both sexes of *Z. jozefienae*.

Stage/Sex	Total bodylength	Prosoma width	Chelicera length	Relative chelicera size to prosoma width
Juvenile	2.31 (0.08)	0.60 (0.02)	0.34 (0.02)	56.7%
Subadult male	2.63 (0.06)	0.85 (0.02)	0.51 (0.01)	60.0%
Subadult female	3.16 (0.07)	0.97 (0.03)	0.59 (0.01)	60.8%
Male	2.74 (0.04)	1.04 (0.03)	0.48 (0.01)	47.1%
Female	4.07 (0.14)	1.23 (0.04)	0.69 (0.02)	56.1%

For each cell n = 10, but for juveniles n = 20.

### Trophic niches in the field

**Figure 2:**
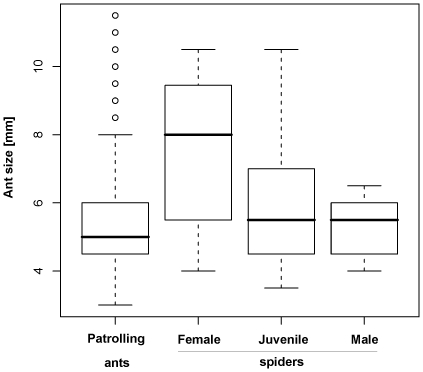
Comparison of the mean ant size captured by *Z. jozefienae* males, females and juveniles (including subadults) and the size of patrolling *Messor barbarus* ants. Thick line is median, boxes are quartiles and whiskers are 1.5-times interquartile range. Points are outliers.

The size of *M. barbarus* workers ranged from 3 to 11.5 mm with medias being the most frequent (65%, n = 100), followed by minor (27%) and major (8%) ant sub-castes. Spiders of different sex and developmental stage captured ants of significantly different sizes(ANOVA, F_3,204_ = 5.9, P = 0.0007, Fig. 2): juveniles (n = 83) and males (n = 5) captured smaller sub-castes than females (n = 20). The average size of ant sub-castes captured by juveniles (5.82±0.18 mm) and males (5.3±0.42 mm) corresponded to the average size of patrolling *Messor* ants (5.84±0.19 mm) (contrasts, P>0.5), while the size of female's prey (7.58±0.23 mm) was significantly larger than the average size of patrolling ants, and also of the prey of males and juveniles (contrasts, P>0.0001, Fig. 2).

**Figure 3:**
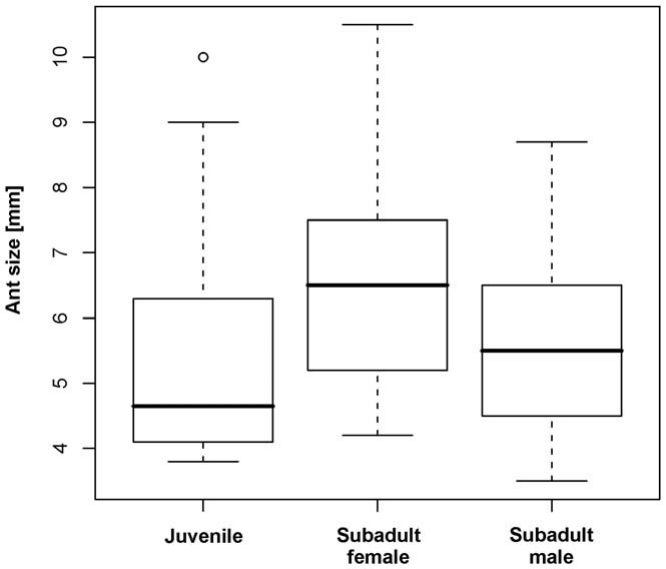
Comparison of the mean size of ant prey (*M. barbarus*) of *Z. jozefienae* juveniles, subadult females and subadult males. Thick line is median, boxes are quartiles and whiskers are 1.5-times interquartile range. Point is outlier.

**Figure 4:**
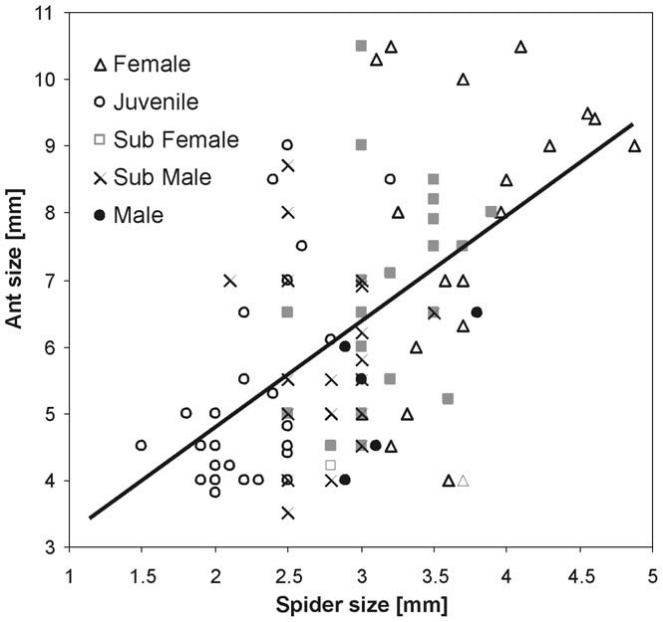
Relationship between the size of ant prey (*M. barbarus*) and the size of *Z. jozefienae* spiders (total body) for all stages and both sexes. The linear model is identical for all stages and both sexes.

Within juvenile spiders, the size of prey differed significantly between recognized ontogenetic stages (ANOVA, F_2,80_ = 4.9, P = 0.01). Subadult females (n = 26) captured significantly larger ants than subadult males (n = 25) and juveniles (n = 32) (contrasts, P<0.01, Fig. 3). The size of captured ants increased linearly with spider body length (Fig. 4). This relationship was similar across all developmental stages and sexes (ANCOVA, F_2,102_ = 0.13, P = 0.88) and is described by a single linear model: *y* = 1.66+1.53*x* (linear regression, F_1,105_ = 39.4, P<0.0001).

The fecundity of wild caught *Z. jozefienae* females ranged between 16 and 32 eggs per egg sac with an average of 24.2 eggs (±1.2). Fecundity was independent of female prosoma width and the total body length (linear regression, F_1,14_<0.31, P>0.6). The size of eggs was on average 0.828±0.018 mm and was independent of the prosoma width and the total body length (LME, F_1,7_<0.09, P>0.37). There was no significant relationship between the clutch size and the egg size (LME, F_1,8_ = 0.2, P = 0.65). The fecundity of laboratory reared females showed a highly significant positive relationship with female body mass before egg laying (linear regression, *y* = −6.484+2363.95*x*, F_1,13_ = 86.9, R^2^ = 0.82, P<0.0001). Again, no significant relationship was found between fecundity and prosoma size (linear regression, F_1,13_ = 0.5, P = 0.48).

### Prey size preference and capture efficiency

**Figure 5:**
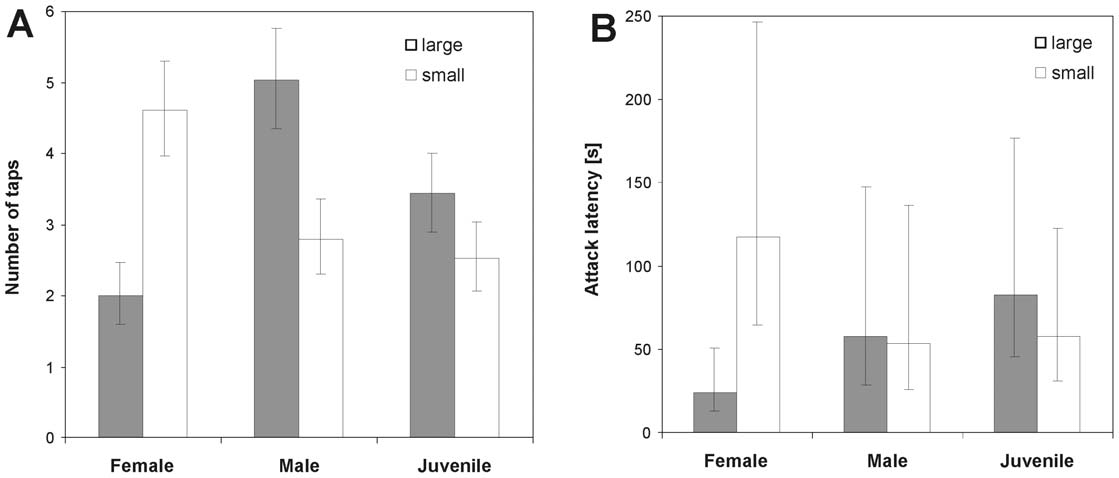
Results of the rey size preference and capture efficiency experiments. A. Comparison of the mean number of taps prior to attack of a small and a large ant (*M. barbarus*) for female, male and juvenile *Z. jozefienae* spiders. B. Comparison of the latency to the first attack of a small and a large ant (*M. barbarus*) for female, male and juvenile *Z. jozefienae* spiders. Whiskers represent 95% confidence intervals for means.

Spiders approached the ant from rear and tapped the dorsal parts of the ant body (legs, gaster or thorax) with the forelegs. The latency to the first tap was similar for small (minor) and large (media or major) ant sub-castes in males, females and juveniles (n = 20 for each, LME, P>0.44). Females tapped large ants significantly fewer times than small ants (LME, F_1,39_ = 14.8, P<0.0001, Fig. 5A). In contrast, males tapped large ants significantly more times than small ants (LME, F_1,38_ = 9.3, P = 0.004). Juvenile spiders did not significantly discriminate small and large ants in tapping frequency (LME, F_1,41_ = 3.2, P = 0.08). Soon after tapping the spider delivered a bite to an ant leg and then retreated to wait for the venom to immobilize the ant at a safe distance. The latency to the first attack differed between males, females and juveniles. In females, latency to attack was significantly shorter for large than for small ants (LME, F_1,35_ = 4.7, P = 0.036, Fig. 5B), while no clear effect of ant size on the latency to the first attack was detected in males and juveniles (LME, P>0.53). Finally, once the ant was immobilized spiders initiated feeding. A majority of spiders, 67% (n = 94), independent of developmental stage and sex (GLM-b, χ^2^_2_ = 1.8, P = 0.4), preferred to feed on the large ant (Exact binomial test, P = 0.001).

**Figure 6:**
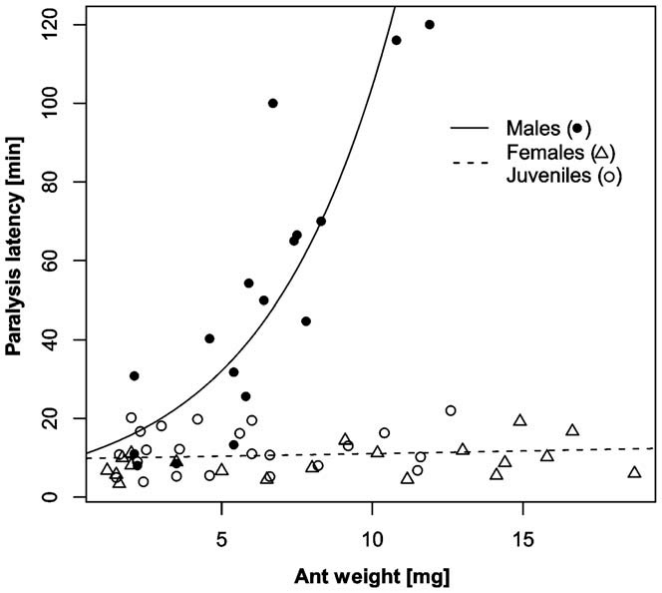
Relationship between the paralysis latency of ants (*M. barbarus*) and their weight for female, male and juvenile *Z. jozefienae* spiders. One model for juveniles and females (*y* = 9.8) and a model for males (*y* = *e* ^2.29+0.24*x*^) are presented.

**Figure 7:**
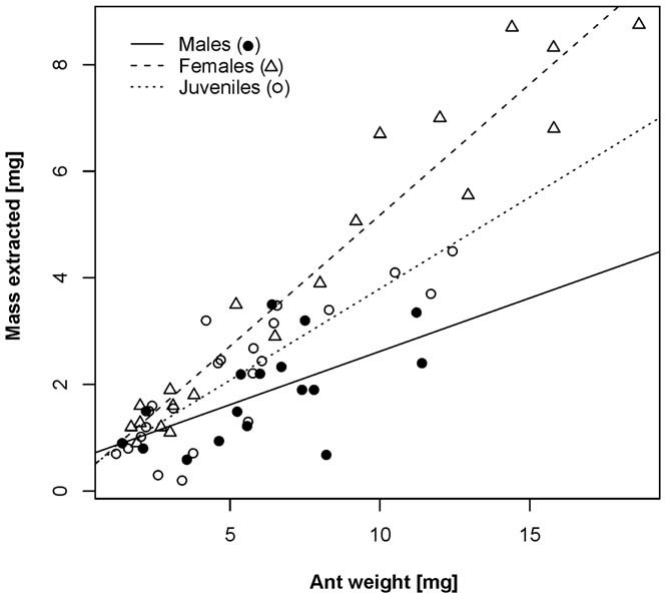
Relationship between the mass extracted and the weight of ants (*M. barbarus*) for male, female and juvenile *Z. jozefienae* spiders. Linear model for females is: *y* = 0.259+0.492*x*; for juveniles *y* = 0.362+0.344*x*; and for males: *y* = 0.619+0.2*x*.

Adults of both sexes and juveniles were able to paralyze *Messor* ants within 2 hours but with significantly different efficiency (GLM-g, F_1,56_ = 5.2, P<0.0001, Fig. 6). In females and juveniles the paralysis latency was more or less similar (contrast, P = 0.05) and relatively constant (approx. 10 min) and independent of ant weight (contrast, P = 0.43). In males, paralysis latency differed significantly from that of females and juveniles (contrasts, P<0.0001) by increasing exponentially with ant weight, hence the larger the ant the longer it took for males to paralyze them. Also feeding efficiency differed between male, female and juvenile spiders (ANCOVA, F_2,54_ = 10.3, P = 0.0002). Females extracted mass at the highest rate which was indicated by a steeper slope which increased linearly with the weight of the ant (Fig. 7). Juveniles extracted mass at 1.4-times lower slope than females (contrast, P = 0.012), and males extracted mass at a 2.5-time lower slope than females (see Fig 7, contrast, P = 0.0001).

## Discussion

We tested predictions of the “sexual selection” and “intersexual niche partitioning” hypotheses underlying sexual size dimorphism and intersexual trophic niche partitioning in a specialist ant predator, the spider *Zodarion jozefienae* in the field. Morphological analyses revealed significant female biased SSD in chelicerae size and body size. These traits were 18–49% larger in females relative to males. Female biased SSD was apparent also in the subadult stage of spiders documenting an ontogenetic development of sexual dimorphism [28]. Field data revealed intersexual trophic niche partitioning in *Z. jozefienae*, with females selectively capturing larger ant sub-castes than males and juveniles, despite a lower frequency of the large ant sub-caste in natural foraging sites. The “sexual selection” hypothesis assumes that males are selected to maximize mating opportunities, while females should maximize fecundity [8], [9], [10]. Fecundity selection should favor female biased dimorphism and predicts a positive relationship between body size/body mass and fecundity [20], [29]. Supporting these predictions, we documented highly female biased SSD in body size and trophic morphology, while fecundity was determined by female body mass but not absolute body size. Furthermore, females displayed preference for large prey and showed a higher relative capture and feeding efficiency compared with males and juveniles. These are traits that likely increase energy intake, which translate into increased offspring production [30]. Males on the other hand, experienced reduced capture efficiency both in time spent hunting and in prey capture efficiency, and they also extracted relatively less mass from prey compared with both females and juveniles. In fact, males dramatically diminished their foraging effort altogether and instead increased mate-searching activities [31]. Our data suggest that intersexual trophic niche partitioning in *Z. jozefienae* may be shaped by higher energy requirements and hence fecundity in females, while the data for males are consistent with a shift from investment in foraging to mating effort in males.

Observations of males in other spider species suggest a similar dramatic change in trophic niche upon maturation to adulthood. For example, *Misumenops* spiders [32] abandon predatory behavior to feed on a plant diet, and males of some web-building spiders give up their capture webs for mate searching and adopt a kleptoparasitic strategy of stealing prey from females [33]. Hence, males appear to allocate resources to foraging in the juvenile and pre-adult stage, and then switch to allocate resources to mate searching and mating in the adult stage.

We note that egg size and fecundity were unrelated to absolute female size (i.e. prosoma width and body length), but instead was highly correlated with body mass. This is a rare example of income breeding among ectotherms [34]. It shows that fecundity selection is acting mainly in the adult stage and is related to energy intake (body weight) rather than absolute body size and potentially balancing selection on female absolute body size. Females likely increase fecundity via preferential selection of the large ant sub-caste and high capture efficiency, and they may additionally gain other ecological benefits from optimizing prey capture efficiency. In the field, we observed that females moved prey of large ant sub-castes to a feeding site away from the ant trails. Ant trails are dangerous feeding sites not only because defending ants can attack *Zodarion* spiders, but also because other generalist predators, such as spiders, scorpions and solifuges, gather at the trails. We observed foraging *Z. jozefienae* spiders on ant trails being preyed upon by solifuges *Gluvia dorsalis* Latreille and spiders (*Thanatus* sp. (Araneae, Philodromidae) and *Nomisia* sp. (Araneae, Gnaphosidae)). The female foraging strategy likely facilitates fewer hunting events that simultaneously increase energy intake and reduce the risk of encounters with potential enemies [35]. Males patrolling the ant trails in search of females would experience increased predation risk, however, the chance of successfully inseminating multiple females would counter early death [36], [37]. In contrast, by reducing the number of foraging bouts females benefit from reducing the risk of encountering predators as their reproductive success depends on survival until eggs have been deposited [38].

Benefits to females of increasing prey capture efficiency and reducing the number of foraging event are factors that should favor adaptations in trophic morphology – the chelicerae - to increase prey capture. The development of chelicerae during ontogenesis revealed strong sex-specific morphological divergence: female chelicerae showed isometric increase with body size, while in contrast, male chelicerae showed significant allometric decrease. Indeed, in females the isometric increase of chelicerae size facilitated the capture of larger prey, as also seen in wolf spiders [22], while this was not the case in males. Generally, the size of captured ants correlated positively with the spider body size. Thus, females switched gradually from minor worker ants to media and major sub-castes during their development, corresponding to their increase in body size and chelicerae size. Males, on the other hand, showed no switch in preference for larger ant sub-castes. Moreover, they appeared to reduce the frequency of prey capture dramatically as only 2% of males were found to capture ants in the field, compared with more than 80% of females and juveniles [31]. These data suggest a shift in allocation of resources in males from foraging in the subadult stage to other activities associated with mate finding in the adult stage.

It is unclear how *Zodarion* spiders recognise ant sub-castes of different sizes. As prey capture occurs overnight eye sight is expected to be of limited importance. The tapping of ant body prior to attack suggests importance of olfactory or tactile stimuli. There might be differences in the cuticle hydrocarbons profiles among sub-castes of *M. barbarus* and *Zodarion* spider might be able to discriminate the sub-castes using chemosensitive hairs situated at the tip of tarsi. This may explain why females tapped smaller ants more than the large ones and males tapped more larger than smaller ants.

An additional prediction from the “sexual selection” hypothesis is that male-biased SSD evolves in traits involved in male-male competition. In spiders, this would result in male-biased SSD in the chelicerae if males used their chelicerae in male-male interactions. Male biased dimorphism in chelicerae size has been documented in the spider *Myrmarachne plataleoides* (O. P.-Cambridge) [39]. Males of this species possess enlarged chelicerae that are used in male-male combats. Interestingly, the chelicerae of *M*. *plataleoides* lack venom ducts in the fangs and are therefore rather useless in prey capture. Consequently, males feed only on small prey items, which results in trophic niche partitioning similar to what we observed in our study. We found no evidence for the use of chelicerae in *Z. jozefienae* as males use little time in male-male combats and use only forelegs [40]. Our study thus indicates that male-male competition is unlikely to shape SSD and trophic niche partitioning in this species.

The “intersexual niche partitioning” hypothesis assumes that trophic dimorphism evolves to reduce competition between the sexes over limited resources, and this hypothesis has received wide support in studies of snakes [16]. In our study system, the ants appear to be an extremely numerous resource as a single nest of *Messor* ants contains thousands of individuals [41]. The density of *Z. jozefienae* spiders appears to be negligible (approx. 10 individuals/nest/day) and hence unable to deplete the ants as a food resource. An adult female is estimated to consume approximately 30 ants during a lifetime (S. Pekár, per. obs.) suggesting no risk of resource depletion. Thus, intersexual competition for ant prey is unlikely. In accordance, we never observed two spiders competing over prey in the field. Interestingly, ants that had been captured and bitten by other spiders and left on the ant trail until complete immobilization as a strategy to reduce the risk of the ant injuring the spider were observed to be kleptoparasitized by adult males [31]. Such opportunistic foraging was suggested to evolve when males invest less in foraging and are less efficient in prey capture, and instead invest time in searching for mates. We found no evidence for competition over ant prey among the sexes, it is therefore unlikely that trophic niche partitioning functions to reduce intersexual resource competition.

We can not rule out that reduced resource competition might be the result of past intersexual niche divergence [6], [8], although it is unlikely in the spider-ant system studied here for the reasons outlined above. Sexual dimorphism in trophic morphology in a direction not predicted by sexual selection might be interpreted as evidence to support the “intersexual niche partitioning” hypothesis [6], [17], and the observed sexual dimorphism with smaller chelicerae in males is theoretically in accordance with this idea. However, as argued above, the data collected in this study are more consistent with sex-specific differences in reproductive strategies, where males upon maturation trade foraging effort with mating effort and hence experience a reduction in chelicerae size.

In this study, we show that trophic niche and SSD in trophic morphology in *Z. jozefienae* spiders correlate with sex-specific reproductive strategies in the adult stage. We propose that intersexual trophic niche partitioning and female biased sexual dimorphism in body size and trophic morphology are shaped primarily by fecundity selection in females, and result from sex-differences in the route to successful reproduction, where females are selected to maximize energy intake and egg production, while males invest in mating effort.

## References

[1] Blanckenhorn WU (2005). Behavioral causes and consequences of sexual size dimorphism.. Ethology.

[2] Delph LF (2005). Processes that constrain and facilitate the evolution of sexual dimorphism.. Am Nat.

[3] Houston D, Shine R (1993). Sexual dimorphism and niche divergence: feeding habits of the Arafura filesnake.. J Anim Ecol.

[4] Castilla AM, Bauwens D, Llorente GL (1991). Diet composition of the lizard *Lacerta lepida* in central Spain.. J Herpetol.

[5] Luiselli L, Capula M, Shine R (1996). Reproductive output, costs of reproduction, and ecology of the smooth snake, *Coronella austriaca*, in the eastern Italian Alps.. Oecologia.

[6] Shine R (1989). Ecological causes for the evolution of sexual dimorphism: a review of the evidence.. Q Rev Biol.

[7] Santos X, González-Solis J, Llorente GA (2000). Variation in the diet of the viperine snake *Natrix maura* in relation to prey availability.. Ecography.

[8] Fairbairn DJ (1997). Allometry for sexual size dimorphism: Pattern and process in the coevolution of body size in males and females.. Annu Rev Ecol Syst.

[9] Trivers R, Campbell B (1972). Parental investment and sexual selection.. Sexual Selection and the Descent of Man 1871-1971.

[10] Andersson M (1994). Sexual Selection..

[11] Serrano-Meneses MA, Szekely T (2006). Sexual size dimoprhism in seabirds: sexual selection, fecundity selection and differential niche-utilisation.. Oikos.

[12] Lindsay WRM, Webster MS, Varian CW, Schwabl H (2009). Plumage colour acquisition and behaviour are associated with androgens in a phenotypically plastic bird.. Anim Behav.

[13] Lindeman PV (2003). Sexual difference in habitat use of Texas map turtles (Emydidae: *Graptemys versa*) and its relationship to size dimorphism and diet.. Can J Zool.

[14] Cox RM, Skelly SL, John-Adler HB (2008). A comparative test of adaptive hypotheses for sexual dimorphism in lizards.. Evolution.

[15] Slatkin M (1984). Ecological causes of sexual dimorphism.. Evolution.

[16] Shine R, Seigel RA, Collins JT (1993). Sexual dimorphism in snakes.. Snakes. Ecology and Behaviour.

[17] Selander RK, Campbell B (1972). Sexual selection and dimorphism in bird.. Sexual Selection and the Descent of Man 1871-1971.

[18] Verwaijen D, Van Damme R, Herrel A (2002). Relationships between head size, bite force, prey handling efficiency and diet in two sympatric lacertid lizards.. Funct Ecol.

[19] Pekár S, Král J, Lubin YD (2005). Natural history and karyotype of some ant-eating zodariid spiders (Araneae, Zodariidae) from Israel.. J Arachnol.

[20] Head G (1995). Selection on fecundity and variation in the degree of sexual size dimorphism among spider species (class Araneae).. Evolution.

[21] Walker SE, Rypstra AL (2001). Sexual dimorphism in functional response and trophic morphology in *Rabidosa rabida* (Araneae: Lycosidae).. Am Midl Nat.

[22] Walker SE, Rypstra AL (2002). Sexual dimorphism in trophic morphology and feeding behavior of wolf spiders (Araneae: Lycosidae) as a result of differences in reproductive roles.. Can J Zool.

[23] Bosmans R (1994). Revision of the genus *Zodarion* Walckenaer, 1833 in the Iberian peninsula and Balearic islands (Araneae, Zodariidae).. Eos.

[24] López JR, Haeger JF (1999). Sequential co-operative load transport in the seed-harvesting ant *Messor barbarus*.. Insectes Soc.

[25] R Development Core Team (2007). http://www.R-project.org.

[26] Pinheiro JC, Bates DM (2000). Mixed-Effects Models in S and S-PLUS..

[27] Crawley MJ (2002). Statistical Computing. An Introduction to Data Analysis using S-Plus..

[28] Badyaev AV (2002). Growing apart: an ontogenetic perspective on the evolution of sexual size dimorphism.. Trends Ecol Evol.

[29] Suter RB (1990). Determinants of fecundity in *Frontinella pyramitela* (Araneae, Linyphiidae).. J Arachnol.

[30] Simpson MR (1995). Covariation of spider egg and clutch size: the influence of foraging and parental care.. Ecology.

[31] Martišová M, Bilde T, Pekár S (2009). Sex-specific kleptoparasitic foraging in ant-eating spiders.. Anim Behav.

[32] Pollard SD, Beck MW, Dodson GN (1995). Why do male crab spiders drink nectar?. Anim Behav.

[33] Schneider JM, Lubin Y (1998). Intersexual conflict in spiders.. Oikos.

[34] Houston AI, Stephens PA, Boyd IL, Harding KC, McNamara JM (2007). Capital or income breeding? A theoretical model of female reproductive strategies.. Behav Ecol.

[35] Brown JS, Kotler BP (2004). Hazardous duty pay and the foraging cost of predation.. Ecol Lett.

[36] Promislow DEL, Montgomerie R, Martin TE (1992). Mortality costs of sexual dimorphism in birds.. Proc R Soc, Biol Sci.

[37] Clutton-Brock TH, Isvaran K (2007). Sex differences in ageing in natural populations of vertebrates.. Proc R Soc, Biol Sci.

[38] Liker A, Szekely T (2005). Mortality costs of sexual selection and parental care in natural populations of birds.. Evolution.

[39] Pollard SD (1994). Consequences of sexual selection on feeding in male jumping spiders (Araneae: Salticidae).. J Zool.

[40] Pekár S (2004). Poor display repertoire, tolerance and kleptobiosis: results of specialization in an ant-eating spider (Araneae, Zodariidae).. J Insect Behav.

[41] Hölldobler B, Wilson EO (1990). The Ants..

